# Venous thromboembolism in patients with acute myeloid leukemia: development of a predictive model

**DOI:** 10.1186/s12959-024-00607-6

**Published:** 2024-04-17

**Authors:** Mirjana Mitrovic, Nikola Pantic, Zoran Bukumiric, Nikica Sabljic, Marijana Virijevic, Zlatko Pravdic, Mirjana Cvetkovic, Nikola Ilic, Jovan Rajic, Milena Todorovic-Balint, Ana Vidovic, Nada Suvajdzic-Vukovic, Jecko Thachil, Darko Antic

**Affiliations:** 1 Clinic of Hematology, University Clinical Center of Serbia, Belgrade, Serbia; 2 Faculty of Medicine, University of Belgrade, Belgrade, Serbia; 3 Faculty of Medicine, Institute for medical statistics and informatics, University of Belgrade, Belgrade, Serbia; 4 Faculty of Medicine, Center for Information and Communication Technologies, University of Belgrade, Belgrade, Serbia; 5 Manchester University NHS, Manchester, Great Britain

**Keywords:** Acute myeloid leukemia, Nomogram, Predictor, Thrombosis, Venous thromboembolism

## Abstract

**Background:**

Patients with acute myeloid leukemia (AML) are at increased risk of venous thromboembolic events (VTE). However, thromboprophylaxis is largely underused.

**Objectives:**

This study aimed to determine possible VTE development risk factors and to develop a novel predictive model.

**Methods:**

We conducted a retrospective cohort study of adult patients with newly diagnosed AML. We used univariate and multivariable logistic regression to estimate binary outcomes and identify potential predictors. Based on our final model, a dynamic nomogram was constructed with the goal of facilitating VTE probability calculation.

**Results:**

Out of 626 eligible patients with AML, 72 (11.5%) developed VTE during 6 months of follow-up. Six parameters were independent predictors: male sex (odds ratio [OR] 1.82, 95% confidence interval [CI]: 1.077–2.065), prior history of thrombotic events (OR 2.27, 95% CI: 1.4–4.96), international normalized ratio (OR 0.21, 95% CI: 0.05–0.95), Eastern Cooperative Oncology Group performance status (OR 0.71, 95% CI: 0.53–0.94), and intensive therapy (OR 2.05, 95% CI: 1.07–3.91). The C statistics for the model was 0.68. The model was adequately calibrated and internally validated. The decision-curve analysis suggested the use of thromboprophylaxis in patients with VTE risks between 8 and 20%.

**Conclusion:**

We developed a novel and convenient tool that may assist clinicians in identifying patients whose VTE risk is high enough to warrant thromboprophylaxis.

## Essentials

Acute myeloid leukemia patients are at increased risk of venous thromboembolism (VTE).

Predictive model for VTE development in acute myeloid leukemia patients was created.

Six parameters were included in the model: male sex, prior history of thrombotic events, international normalized ratio (iNR), Eastern Cooperative Oncology Group performance status and intensive therapy approach.

This model could identify patients whose VTE risk is high enough to warrant thromboprophylaxis.

## Background

Thrombosis is one of the most prominent causes of morbidity and mortality among patients with cancer [1–4]. The risk of thrombosis in acute leukemias might be similar to or even higher than the risk for those with solid neoplasms and varies between 2 and 22% [5–9]. This heterogeneity could be related to numerous factors, such as the different acute leukemia types, type of central venous line (CVL), frequency of CVL insertion, and type of chemotherapy (all-trans-retinoic acid, L asparaginase) [5–9].

The high incidence of VTE in acute leukemias raises the question of whether primary VTE thromboprophylaxis is needed to prevent this complication. Nevertheless, the wide application of thromboprophylaxis is limited by a very high prevalence of thrombocytopenia and perceived high risk of bleeding, as well as the lack of evidence-based guidelines to assist clinicians [10]. Consequently, determining VTE development risk factors in patients with acute leukemias will allow clinicians to risk-stratify patients and individualize patient surveillance and anticoagulant prophylaxis.

A correlation between epidemiologic, genetic, and molecular features of acute leukemias and thrombosis has rarely been assessed. Most studies included all types of acute leukemias, with conflicting and inconsistent results. Factors that were identified as predictive for thrombosis were: male sex, age, comorbidities, acute leukemias type, CVL insertion, previous thrombosis, platelet count (cut-off values varied from > 50 × 10^9^/L to > 350 × 10^9^/L), D-dimer level, disseminated intravascular coagulation (DIC), normal karyotype, FLT3-ITD and NPM1 mutations, and intermediate/high-risk cytogenetics [5–9, 11–15]. A valid risk assessment model (RAM) should ideally be used to make decisions regarding anticoagulation treatment for the prevention of cancer-associated VTE in acute leukemias. Various models to predict the risk of VTE development in oncological patients have been constructed [16]. However, these models were suboptimal, not applicable, or not assessed in patients with acute leukemias [13–16].

This study aimed to assess potential risk factors and predictive biomarkers for VTE development in newly diagnosed patients with acute myeloid leukemia (AML) and develop a convenient predictive model hinged on patient- and disease-associated parameters.

## Methods

In this retrospective cohort study, we included all adults (≥ 18 years of age) with a newly diagnosed AML who were diagnosed and treated in the Clinic for Hematology at the University Clinical Center of Serbia between January 2009 and December 2021. The retrieval of information and publication of these results were approved by the Institutional Review Board of the University Clinical Center of Serbia (protocol number III 41,004).

AML diagnoses were confirmed using cytological, flow cytometry, and cytogenetic findings according to the World Health Organization and European Leukemia Net criteria [17, 18]. Patients with acute promyelocytic leukemia were excluded. Participants were followed from the time of diagnosis to VTE development, death, or 6 months after the diagnosis. Patients were treated in an intensive (“3 + 7” induction followed by intermediate-dose cytarabine [IDAC] consolidation and allogenic hematopoietic stem cell transplantation), non-intensive (azacytidine, low-dose chemotherapy), or supportive manner [18, 19]. The treatment protocol was selected according to the patient’s age, Eastern Cooperative Oncology Group performance status (ECOG PS), Hematopoietic Cell Transplantation-specific Comorbidity Index, and disease risk [18, 19]. CVL was placed in all patients who were treated intensively. During the study period, we used nontunneled, Arrow two lumen CVL (Teleflex, Morrisville, North Carolina.

Data collection included: demographic factors (age, sex), body mass index (BMI), smoking status, comorbidities (including previous thrombosis), concomitant therapy, ECOG PS, Hematopoietic Cell Transplantation-specific Comorbidity Index, baseline laboratory findings (complete blood count, fibrinogen, prothrombin time [PT], International Normalized Ratio [INR], activated partial thromboplastin time [APTT], D-dimer, lactate dehydrogenase [LDH], leukemia-related parameters (cytogenetics, molecular genetics [FLT3, NPM1], flow cytometry), type (intensive, non-intensive, palliative therapy) and phase of leukemia-related therapy, the presence of a CVL, Khorana and Al Ani scores, and concurrent COVID-19 positivity. DIC was diagnosed according to the International Society on Thrombosis and Haemostasis (ISTH) scoring system [20]. All laboratory parameters, as well as comorbidities, concomitant therapy, and smoking status, were assessed on the day of diagnosis or the nearest day before, in the time frame of 3 days.

The primary outcome was the appearance of symptomatic imaging-confirmed VTE, including upper and lower limb deep venous thrombosis (DVT), pulmonary embolism (PE), thrombosis of unusual sites (cerebral and portal vein thrombosis), and symptomatic CVL-related thrombosis (DVT of any localization related to the presence of a CVL). DVT diagnosis required compression ultrasound evidence of a thrombus. Acute PE was defined as the presence of filling defects on computed tomography pulmonary angiography.

### Statistical approach

To develop a clinical prediction model, at least 5–10 events for each predictor were needed. Therefore, based on our center’s previous data, where 11.4% of patients with AML developed VTE, a population of 600 patients was considered sufficient for exploring the predictive model [21]. In statistical analysis, we used all eligible cases without imputation.

The statistical analysis was performed using the IBM SPSS Statistics 22 software package (SPSS Inc., Chicago, IL, USA) or R software environment (R Core Team, 2021). Categorical variables were presented as absolute or relative frequencies and were compared with the Chi-square or Fisher’s exact test where appropriate. The normality of the distribution was assessed using the Kolomogrov–Smirnov test and histogram. Continuous variables with normal distribution were presented as mean and standard deviation (mean ± SD), whereas non-normally distributed variables were presented as median and range. Mann–Whitney U test or t-test was used to compare groups for continuous variables. The significance level was set at *p* < 0.05.

Univariate and multivariable logistic regression analyses were used to calculate and validate the risk factors for thrombosis development. Variables that were significant in the univariate model were included in the multivariable logistic regression analysis. Multicollinearity was inspected by studying VIF, and VIF > 4 was considered unacceptable. The nomogram was constructed in accordance with the results of the final multivariable logistic regression to depict the individual probabilities of VTE in patients with AML. Discrimination was assessed via a receiver operating characteristic (ROC) curve, and the area under the curve (AUC/C statistics) was used to quantitatively express the ability of the nomogram to predict VTE in patients with AML. A C statistics value of 0.5 indicates that outcomes are completely random, whereas a C statistics value of 1 indicates that the model is a perfect predictor. The calibration of the nomogram was represented using a calibration curve where actual and predicted probabilities were compared and assessed by calibration slope (1.000) and calibration intercept (0.000). Finally, decision curve analysis was used to express the potential clinical value of the risk prediction model. Model performance was evaluated using the “ABCD approach” referenced in the TRIPOD literature [22]. Nonparametric bootstrapping was used for internal validation to get bias-corrected estimates [23, 24]. For demonstration of the selection of statistical methods, a Cox hazard model was also constructed wherein death was considered a censoring event and not a competing risk and included the same specification as the competing risks model.

## Results

### Patients and disease characteristics

In total, 626 consecutive patients with de novo AML were treated at our center between January 2009 and December 2021. The mean age of the participants was 55 (range: 18–81) years, and 348 (55.6%) were males. Among the 626 patients, intensive chemotherapy was delivered in 462 (73.8%). A prior history of thrombotic events was recorded in 46 (7.3%) patients; most of them had arterial thrombotic events (AIM 26, CVI 12). At the time of hospitalization, 47 (7.5%) patients were on anticoagulant therapy, and 31/626 (4.9%) were on antiplatelet therapy. Patients and disease characteristics are shown in Table 1.

### Venous thromboembolic events and bleeding

During the 6-month follow-up, 72/626 (11.5%) patients developed VTE: CVL-related thrombosis in 55/72 (76.4%), DVT in 13/626 (20.8%), and PE in 4/626 (0.007%). Most frequently, VTE was diagnosed during induction (34/72, 47.2%), whereas before therapy, during consolidation, during transplantation, and at the time of relapse, VTE was recorded in 4/72 (5.6%), 27/72 (37.5%), 3/72 (4.2%), and 4/72 (5.5%) patients, respectively. The median time to thrombosis was 3 (range: 0.03–24) months.

**Fig. 1:**
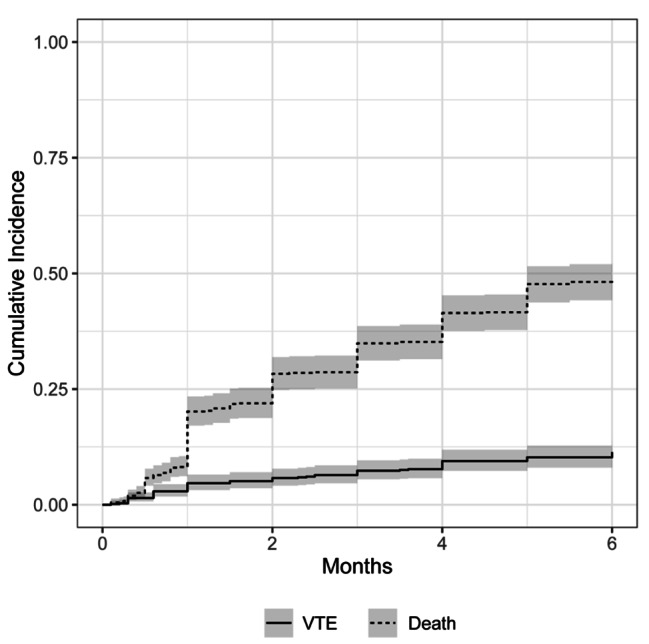
Cumulative incidence of venous thromboembolism and death with confidence interval

The 6 months cumulative incidence of thrombosis using Kaplan–Meier method was 15.9% (95% CI 12.3%-19.4%) in comparison to 11.4% (95% CI, 9.0–14.0%) in the competing risk framework (Fig. 1). 1.

During 6 months of follow-up, bleeding events were registered in 260/626 (41.5%) patients, with grades 3 and 4 in 83/626 (13.3%) patients. Major bleeding events were not registered during anticoagulant therapy. VTE development (Hazards ratio [HR] 0.525, 95% CI: 0.395–0.698, *p* < 0.001) and better performance status (HR 1.251, 95% CI: 1.141–1.372, *p* ≤ 0.001) were predictors of longer survival.

### Development of the prediction score

Among the 38 tested parameters, 6 were predictors of VTE: male sex (odds ratio [OR] 1.82, 95% CI: 1.08–2.06, *p* = 0.025), prior history of thrombotic events (OR 2.27, 95% CI: 1.04–4.96, *p* = 0.001), INR (OR 0.21, 95% CI: 0.05–0.95, *p* = 0.043), ECOG PS (OR 0.71, 95% CI: 0.53–0.94, *p* = 0.017), CVL (OR 3.88, 95% CI: 1.38–10.89, *p* = 0.010), and intensive therapy (OR 2.05, 95% CI: 1.07–3.91, *p* = 0.030). Comparisons of patient and disease parameters are shown in Table 1. The C statistics value for the Al-Ani model was ROC = 0.551.

**Table 1:** Comparison of patients with and without thrombosis according to demographic and clinical parameters

Parameter			All (*n* = 626)	Missing values (%)	Patients with thrombosis (*n* = 72)	Patients without thrombosis (*n* = 554)	OR	95% CI	*p*-value	
Age (years)*			55.1 ± 13.4	0	52.9 ± 13.7	55.4 ± 13.3	0.99	0.97–1.004	0.137	
Male sex (%)			348 (55.6)	0	49 (68.1)	299 (54.0)	1.82	1.08–2.07	0.025	
Smokers (%)			277 (46.8)	16	35 (51.5)	242 (46.2)	1.24	0.75–2.05	0.412	
BMI^1^ *			25.3 ± 4.7	7.7	25.6 ± 4.0	25.2 ± 4.8	1.01	0.96–1.07	0.598	
Prior history of thrombotic events (%)			42 (6.8)	12.5	9 (12.7)	33 (6.9)	2.27	1.04–4.96	0.041	
ECOG PS^2^ (%)		0	102 (16.7)	13.3	17 (25.0)	85 (15.7)	0.71	0.53–0.94	0.017	
		1	256 (41.9)		30 (44.1)	226 (41.6)				
		2	182 (29.8)		17 (25.0)	165 (30.4)				
		3	48 (7.9)		3 (4.4)	45 (8.3)				
		4	23 (3.8)		1 (1.5)	22 (4.1)				
Comorbidities		Total number **	1 (0–7)	1.9	1 (0–4)	1 (0–7)	0.85	0.67–1.08	0.193	
		Diabetes (%)	102 (17.4)	17.4	10 (14.5)	92 (17.8)	0.78	0.39–1.59	0.498	
		Hypertension (%)	156 (25.0)	17.4	14 (20.3)	142 (27.5)	0.67	0.36–1.25	0.208	
Antiplatelet therapy (%)			33 (5.4)	1.9	5 (7.1)	28 (5.1)	0.71	0.26–1.89	0.488	
HCT CI^3^ (%)			1 (0–9)	2.4	1 (0–4)	1 (0–9)	0.83	0.83 − 0.69	0.052	
Khorana score (%)		0	112 (17.9)	11.5	13 (18.1)	99 (17.9)	0.94	0.66–1.32	0.708	
		1	322 (51.4)		39 (54.2)	283 (51.1)				
		2	184 (29.4)		19 (26.4)	165 (29.8)				
		3	8 (1.3)		1 (1.4)	7 (1.3)				
Al Ani score (%)		0	317 (50.6)	0	30 (41.7)	287 (51.8)	1.26	0.90–1.78		0.185
		1	296 (47.3)		40 (55.6)	256 (46.2)				
		2	0 (0.0)		0 (0.0)	0 (0.0)				
		3	8 (1.3)		2 (2.8)	6 (1.1)				
		4	5 (0.8)		0 (0.0)	5 (0.9)				
COVID-19 (%)			59 (9.4)	11.6	7 (9.7)	52 (9.4)	1.04	0.45–2.38		0.931
^4^CNS involvement (%)			54 (20.5)	-	11 (30.6)	43 (18.9)	1.89	0.87–4.14		0.110
^5^WBC (normal: 3.6–10 × 10^9^/L) **			9.8 (0.4-473.2)	0	10.5 (0.7-211.6)	9.7 (0.4-473.2)	0.998	0.993–1.002		0.321
Platelet count (normal:150–400 × 10^9^/L)**			49 (1-726)	0	56 (1-220)	47 (1-726)	1.001	0.998–1.004		0.370
Hemoglobin (normal: 120–160 g/L)*			95.8 ± 17.8	0	97.0 ± 18.8	95.7 ± 17.4	1.004	0.991–1.018		0.542
^6^LDH (normal, 220–460 U/L)**			458 (105–8902)	9.4	384 (180–4150)	465 (105–8902)	1.000	0.999-1.000		0.170
Fibrinogen (normal: 2.2–5.5 g/L)**			5.4 (0.3–56.0)	5.2	5.6 (1.4–8.5)	5.3 (0.3–56.0)	0.928	0.821–1.048		0.229
INR (normal: 0.8–1.3%)*			1.22 ± 0.19	5.2	1.18 ± 0.17	1.23 ± 0.20	0.21	0.05–0.95		0.043
^8^APTT (normal: 25.1–36.5 s)*			29.2 ± 5.6	5.2	28.4 ± 4.2	29.3 ± 57	0.96	0.91–1.02		0.198
D dimer (normal: 0–0.5 µg/L)**			2.5 (0.1–158.0)	26.5	2.1 (0.3-100.8)	2.5 (0.1–158.0)	0.99	0.98–1.01		0.649
^9^ISTH DIC score ≥ 5 (%)			131 (41.3)	26.5	12 (28.6)	119 (43.3)	0.52	0.26–1.07		0.075
Blast peripheral blood (%)			16 (0–99)	-	15 (0–98)	17 (0–99)	0.99	0.98–1.003		0.182
FAB (%)	0		32 (5.3)	3.4	3 (4.3)	29 (5.4)	0.99	0.90–1.10		0.881
	1		69 (11.4)		12 (17.4)	57 (10.6)				
	2		150 (24.8)		19 (27.5)	131 (24.4)				
	3		2 (0.3)		0 (0.0)	2 (0.4)				
	4		172 (28.4)		16 (23.2)	156 (29.1)				
	5		99 (16.4)		7 (10.1)	92 (17.2)				
	6		2 (0.3)		0 (0.0)	2 (0.4)				
	7		1 (0.2)		0 (0.0)	1 (0.2)				
	9		78 (12.9)		12 (17.4)	66 (12.3)				
^10^ELN classification (%)		Good	66 (11.4)	-	8 (11.9)	55 (11.3)	0.88	0.58–1.33		0.529
		Intermediate	330 (59.5)		42 (62.7)	288 (59.0)				
		High	162 (29.2)		17 (25.4)	145 (29.7)				
^11^FLT3 ITD positivity (%)			63 (19.9)	-	9 (20.9)	54 (19.7)	1.08	0.49–2.38		0.852
^12^NPM1 positivity (%)			59 (24.4)	-	11 (33.3)	48 (23.0)	1.68	0.76–3.70		0.201
^13^CD56 positivity (%)			175 (33.1)	22.3	19 (29.7)	156 (33.5)	0.84	0.47–1.48		0.539
CD13 positivity (%)			510 (93.1)	22.3	59 (90.8)	451 (93.4)	0.7	0.28–1.74		0.440
CD34 positivity (%)			382 (69.5)	22.3	42 (64.6)	340 (70.1)	0.78	0. 45-1.34		0.368
CD33 positivity (%)			512 (93.1)	22.3	60 (90.9)	452 (93.4)	0.71	0.28–1.76		0.458
CD117 positivity (%)			482 (87.8)	22.3	55 (87.8)	427 (87.9)	0.95	0.43–2.09		0.899
CD7 positivity (%)			126 (23.8)	22.3	11 (17.5)	115 (24.6)	0.65	0.33–1.28		0.213
CD15 positivity (%)			178 (34.0)	22.3	21 (33.9)	157 (34.0)	0.99	0.57–1.74		0.986
CD19 positivity (%)			49 (9.5)	22.3	6 (9.8)	43 (9.5)	1.04	0.42–2.56		0.932
^14^CVL inserted (%)			519 (82.9)	11.5	68 (94.4)	451 (81.4)	3.88	1.38–10.89		0.010
Therapy type (%)		Intensive	453 (72.4)	11.3	60 (83.3)	393 (70.9)	2.05	1.07–3.91		0.030
		Non-intensive	173 (27.6)		12 (16.7)	161 (29.1)				

^1^BMI, body mass index; ^2^ECOG PS, Eastern Cooperative Oncology Group performance status; ^3^HCT CI, Hematopoietic Cell Transplantation-specific Comorbidity Index; ^4^CNS, central nervous system; ^5^WBC, white blood cells; ^6^LDH, lactate dehydrogenase; ^7^PT, prothrombin time; ^8^aPTT, activated partial thromboplastin time; ^9^ISTH DIC score, International Society on Thrombosis and Hemostasis disseminated intravascular coagulation score; ^10^ELN, European Leukemia Net; ^11^FLT3 ITD, internal tandem duplication in FMS-like tyrosine kinase 3 gene; ^12^NPM1, nucleophosmin 1; ^13^CD, cluster of differentiation; ^14^CVL, central venous line *mean ± standard deviation ** median (range)

Since CVL persistence and therapy type were interconnected, we decided to include sex, prior history of thrombotic events, PT, ECOG PS, and therapy type in the predictive model (Table 2).

**Table 2:** Multivariable logistic regression model with venous thromboembolic event as dependent variable

Analyzed variable	B	*P*	OR	95% CI
Sex (male vs. female)	0.739	0.011	2.093	1.188–3.689
Previous thrombosis	0.991	0.018	2.695	1.187–6.117
ECOG PS	-0.293	0.070	0.746	0.544–1.024
INR	-1.377	0.113	0.252	0.046–1.386
Therapy type (intensive vs. non-intensive)	0.383	0.278	1.467	0.734–2.932

ECOG PS, Eastern Cooperative Oncology Group performance status; INR, International Normalized Ratio

Table 3 demonstrates the bias introduced using Cox PH models in the presence of competing risks.

**Table 3:** Results of competing risks model compared to the Cox PH model

Analyzed variable	Competing Risks		Cox	
	sHR (95% CI)	*p*-value	HR (95% CI)	*p*-value
Sex (male vs. female)	1.92 (1.14–3.23)	0.014	1.84 (1.08–3.14)	0.024
Previous thrombosis	2.39 (1.20–4.77)	0.013	2.25 (1.11–4.56)	0.025
ECOG PS	0.75 (0.56–1.01)	0.056	0.83 (0.62–1.11)	0.203
iNR	0.28 (0.05–1.68)	0.160	0.38 (0.08–1.89)	0.239
Therapy type (intensive vs. non-intensive)	1.33 (0.70–2.55)	0.390	1.16 (0.61–2.22)	0.652

*HR hazard ratio, sHR sub distribution hazard ratio

**Fig. 2:**
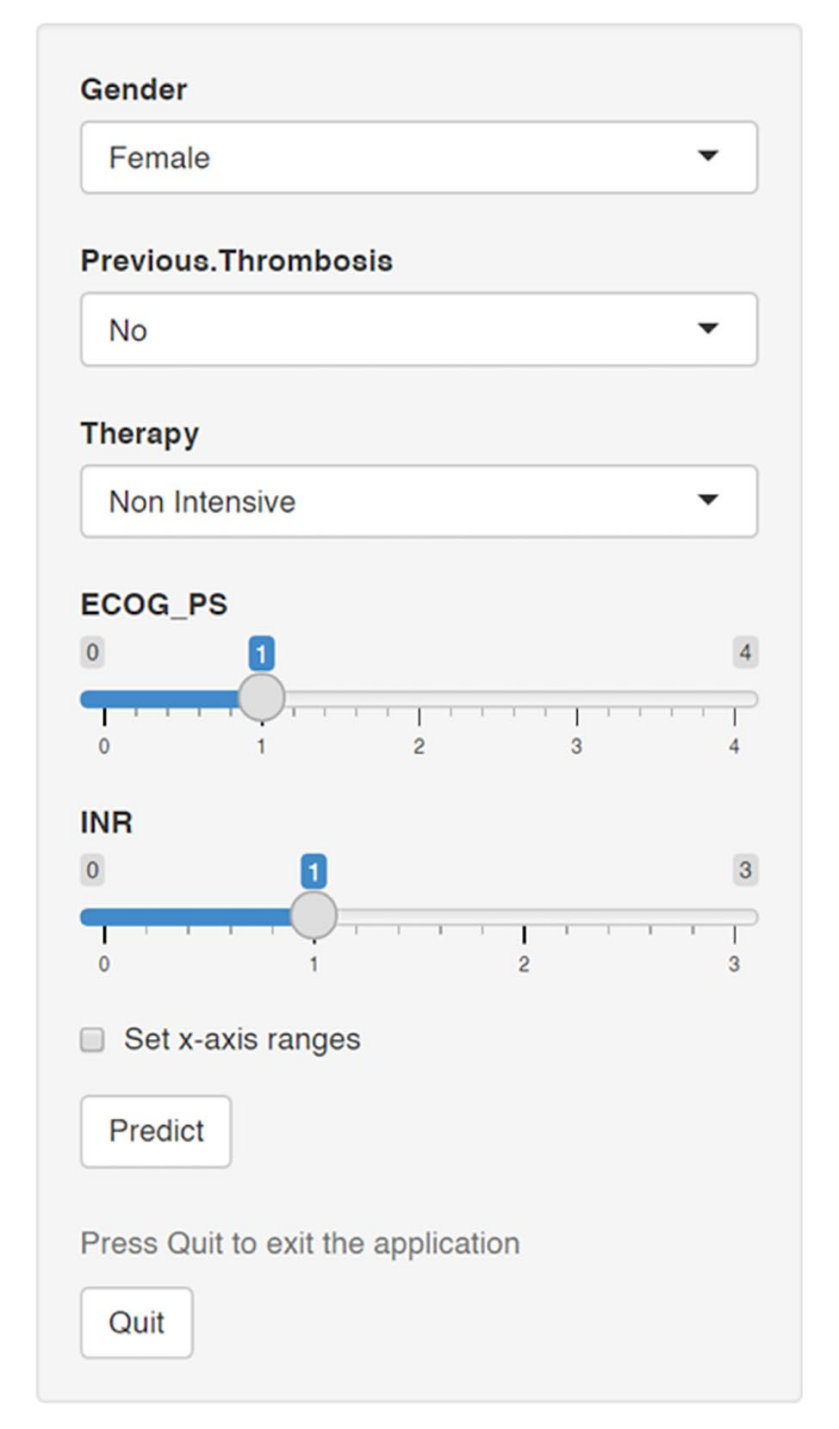
Nomogram for venous thromboembolic event development. DVT, deep venous thrombosis; ECOG PS, Eastern Cooperative Oncology Group performance status; HCT CI, Hematopoietic Cell Transplantation-specific Comorbidity Index; PT, prothrombin time

Figure 2 shows the nomogram that integrated all the predictors in the last multivariable model. This nomogram depicts the relative contribution of every prognostic parameter to the final score and the weight of factors regarding the probability of VTE development. Link to the nomogram calculator: https://kizostat.shinyapps.io/VTE23/.

**Fig. 3:**
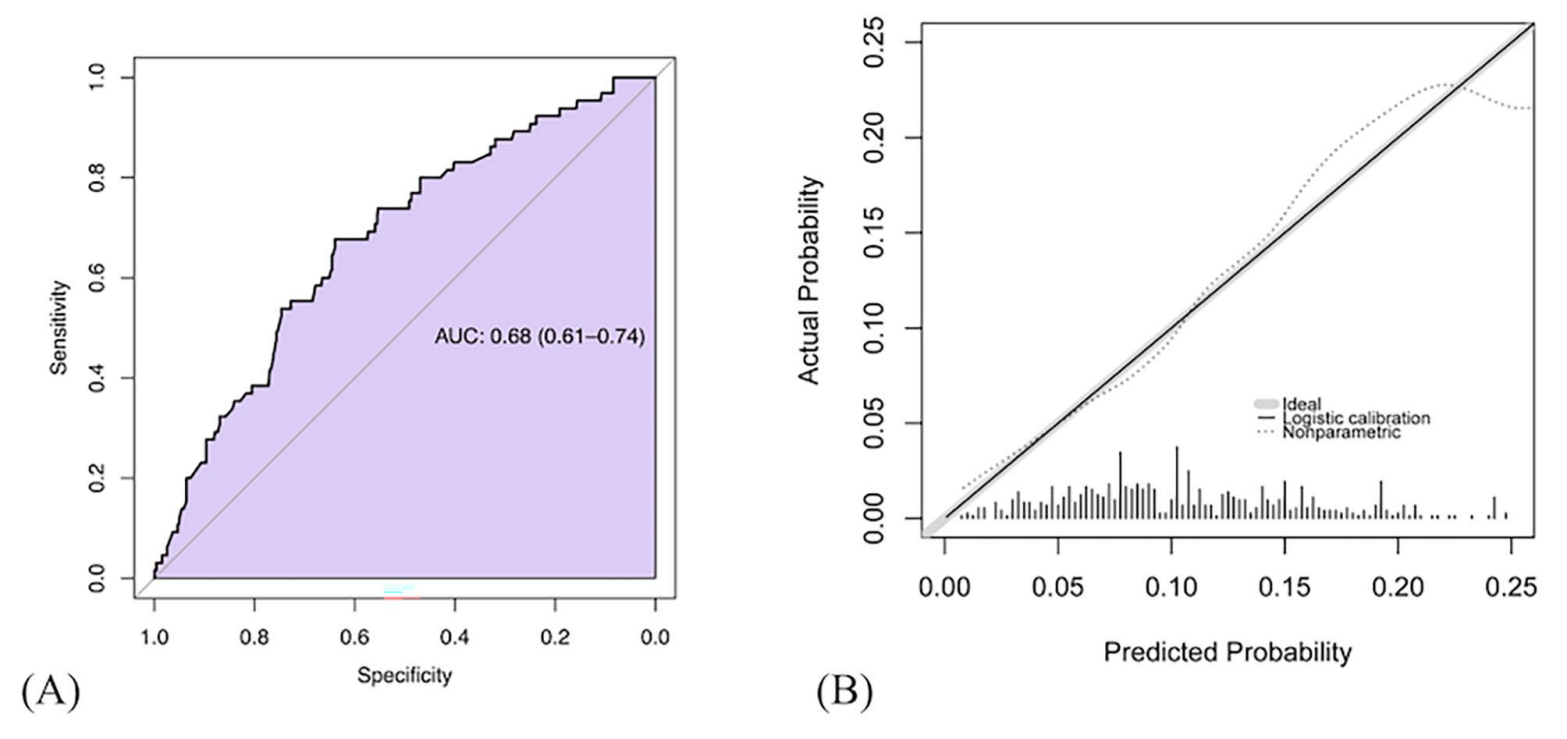
(**A**) Prediction performance and (**B**) cross-validated calibration plots of the clinical prediction model

The discrimination and prognostic capacity of the nomogram were illustrated using the ROC curve (Fig. 3A). The C statistics value for the model was 0.68 (95% CI: 0.61–0.74), which indicated satisfactory accuracy. The model was adequately calibrated, with no indication of systematic underestimation or overestimation of VTE in patients with AML. Bootstrapping for internal validation is represented in Fig. 3B.

**Fig. 4:**
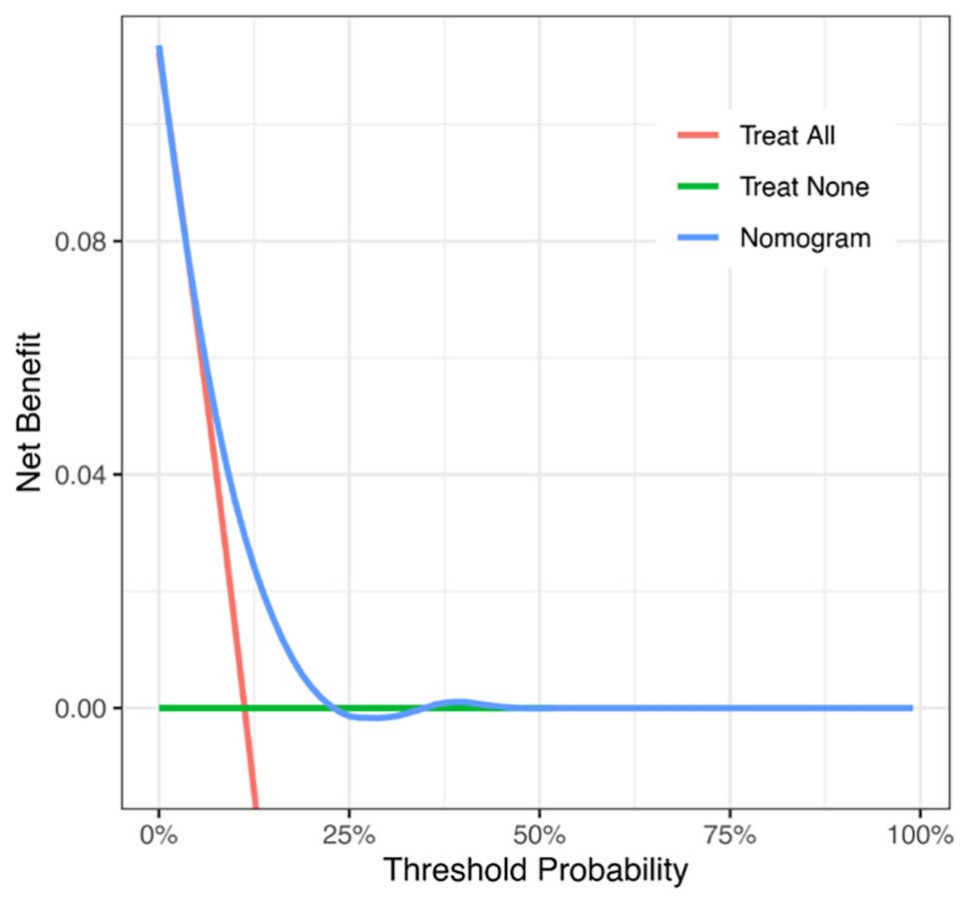
Decision-curve analysis for primary thromboprophylaxis in AML. The probability threshold represents the predicted risk of venous thromboembolism in AML for recommending primary thromboprophylaxis. The net clinical benefit balances the risk of venous thromboembolism with the potential harms of unnecessary thromboprophylaxis, which was calculated as the true-positive rate minus the weighted false-positive rate

Finally, the decision-curve analysis revealed the usefulness of the nomogram for deciding in which patients thromboprophylaxis should be used (Fig. 4). Notably, a benefit for thromboprophylaxis was observed in patients with thromboembolism risks in the range of 8–20% (Fig. 4). For the threshold probability of 10%, the net benefit was 0.034 compared to 0.014 for the treatment all or none.

## Discussion

We developed a novel, easy-to-use clinical prediction model for VTE in adult patients with AML. Analysis of numerous parameters generated a model that included the patient’s sex, history of previous thrombosis, comorbidity score, ECOG PS, INR value, and intensive therapy approach. Our nomogram had the ability to separate patients with and without thrombosis during AML therapy. Decision-curve analysis showed that the use of our model to determine thromboprophylaxis utility could reduce the risk of VTE compared with applied thromboprophylaxis in an all or none approach. The results of our competing risk analysis did not show a notable impact of death.

Our study investigated the predictive value of 38 disease-, therapy-, and patient-related parameters for VTE development. The predictive risk factors observed in our study were consistent with those reported in previous studies. Patients with a prior history of thrombosis in our group had a more than two-fold higher risk of recurrence, which is in line with data from studies in the general population. A higher rate of prior VTE has been associated with an increased recurrence rate in patients with cancer-associated thrombosis, acute leukemias, and particularly AML [6, 9, 21, 25, 26]. Male patients in our study had a two-times higher risk for thrombosis development compared with women. Interestingly, some studies showed a slightly higher risk of VTE in men than in women, whereas others showed that the incidence was higher in women than in men [27, 28]. The Tromso study reported a slightly higher overall VTE rate in women. However, the incidence was higher in women up to 60 years of age, and thereafter, it became slightly higher in men [29]. High VTE incidence in women can be explained by the use of hormonal contraceptives and by pregnancy in this phase of life. However, when the results were adjusted for the aforementioned factors, men were twice as likely as women to develop VTE, which was also observed in our study [30].

ECOG PS is used regularly as a tool for choosing therapy types for patients with AML [18, 19]. The ECOG PS has demonstrated its predictive value for VTE development and overall survival in cancer patients [31–34]. Generally, patients with high ECOG do not qualify for intensive therapy. Interestingly, to the best of our knowledge, this parameter has not been investigated as a predictor of VTE in patients with acute leukemias. In our study, lower ECOG PS was a predictor of longer overall survival and, surprisingly, a predictor of VTE development. Both results could be explained by the longer survival of patients with low scores and, consequently, a higher number of hospitalizations, CVL insertions, and intensive chemotherapies.

The predictive value of therapy type (intensive vs. supportive) for DVT in patients with AML was not assessed. In our group, patients treated with intensive therapy developed thrombosis more frequently. Intensive chemotherapy can increase the risk of thrombosis via direct endothelial damage, destruction of leukemic cells releasing thrombogenic substances, and reduced synthesis of natural anticoagulants due to liver damage [35–38]. According to our study and studies conducted in the general population, in patients with AML, CVL insertion is a proven risk factor for thrombosis development [39–42]. Considering that intensive treatment requires CVL insertion and that chemotherapy can be thrombogenic, we decided to include the therapy type in our final model.

Data regarding the predictive value of the DIC score according to the ISTH criteria are conflicting. Libourel et al. showed that DIC at the time of AML diagnosis was associated with an increased VTE rate. Moreover, PT, antithrombin, and D-dimer were marked as individual predictors [43]. Higher D-dimer levels are a widely used biomarker for DVT prediction in patients with cancer [44, 45]. In contrast, Martella et al. failed to prove the predictive value of ISTH DIC score, PT, or D-dimer levels [9]. In their study, as well as in a study by Al-Ani et al., high platelet counts were predictive [6, 9]. We calculated the ISTH DIC score at the time of diagnosis and tried to validate its potential to predict the risk of VTE in AML, but we did not see any significant association. The only coagulation-related parameter shown to be predictive was PT (INR).

Although the use of prophylactic anticoagulation is not uncommon in patients with AML, the evident bleeding risk could outweigh its clinical benefit [10]. Therefore, developing a model for adequate risk assessment would be useful. The new International Initiative on Thrombosis and Cancer guidelines recommend thromboprophylaxis for patients with cancer at high risk for VTE development and low risk for bleeding [46]. Several RAMs have been developed for VTE risk assessment in oncology [16]. Among them, the Khorana score is the most commonly used. However, patients with AL were excluded from the original study, and the score was insufficient in later studies conducted on patients with AML [6, 13]. Other RAMs widely used in patients with cancer were not applicable or not assessed in patients with acute leukemias. The Al-Ani et al. scoring model, the only RAM model developed for thrombosis prediction in patients with acute leukemias, consists of three components: prior history of VTE, acute leukemia type, and platelet count [6]. Further, the score was developed for all acute leukemia types, diseases with different VTE and DIC frequencies, and different therapy types. These can lead to a high possibility of prophylactic anticoagulant therapy overtreatment or undertreatment in some patient groups. To the best of our knowledge, our study was the first validation study of the Al Ani score. Unfortunately, the score was not predictive in our group of patients with AML.

Our second goal was to set risk thresholds for prophylactic anticoagulation treatment. The American College of Clinical Pharmacy guidelines recommend long-term anticoagulation therapy in patients with VTE with a recurrence risk higher than 10% at 12 months [47]. A model developed by Pabinger et al. posited that thromboprophylaxis was justified for patients with cancer who had a predicted 6-month risk of VTE development of ≥ 15% [45]. Our model showed a positive net benefit for probability thresholds between 8 and 20%. For example, if the clinician wants to use a nomogram to decide on whether to use anticoagulation in a specific patient with a personal threshold of 10%, the net benefit would be 0.034, which is superior to the treatment-all of 0.014 and treatment-none. However, the risk threshold for prophylactic anticoagulation treatment has to be tested prospectively.

This study had some limitations. First, as this is a retrospective study, the underlying bias cannot be overcome. Second, our hospital is an academic center, mainly treating patients qualified for intensive therapy; therefore, our population probably does not reflect the heterogeneous AML population. Third, our group of patients included patients with an indication for anticoagulant and antiplatelet therapy which can contribute to bias. Fourth, mostly catheter-related thromboses occur in our center. Notably, CVL-associated thrombosis has a different pathogenesis and treatment than DVT or PE, and the risk of recurrence is also low. Further, the frequency of CVL-related thrombosis can vary between centers due to the use of different CVL types, materials, and lumens. Conversely, CVL-associated thrombosis complications can include loss of venous access with delay in treatment, infection, post-thrombotic syndrome, and rarely PE. An external validation of our study, which is crucial, is in progress.

Finally, bleeding is a very frequent complication during AML treatment, with 13.3% of patients who developed grade 3 or 4 bleeding events during follow-up in our group. Consequentially, the inherent risk of bleeding may offset the benefits of prophylactic anticoagulation therapy. Therefore, tools for the assessment of bleeding risk in AML are needed together with RAM for thrombosis development.

## Conclusions

We developed a novel and simple tool to assist clinicians with identifying those patients with AML who might benefit from thromboprophylaxis. Our tool can also determine patients with unsubstantial risk of VTE, in whom the increased risk of bleeding due to thromboprophylaxis would outweigh the benefits. However, dedicated primary thromboprophylaxis trials in patients with acute leukemias in a risk-adapted manner are also required.

## Data Availability

the datasets used and analyzed during the current study are available from the corresponding author on reasonable request.
